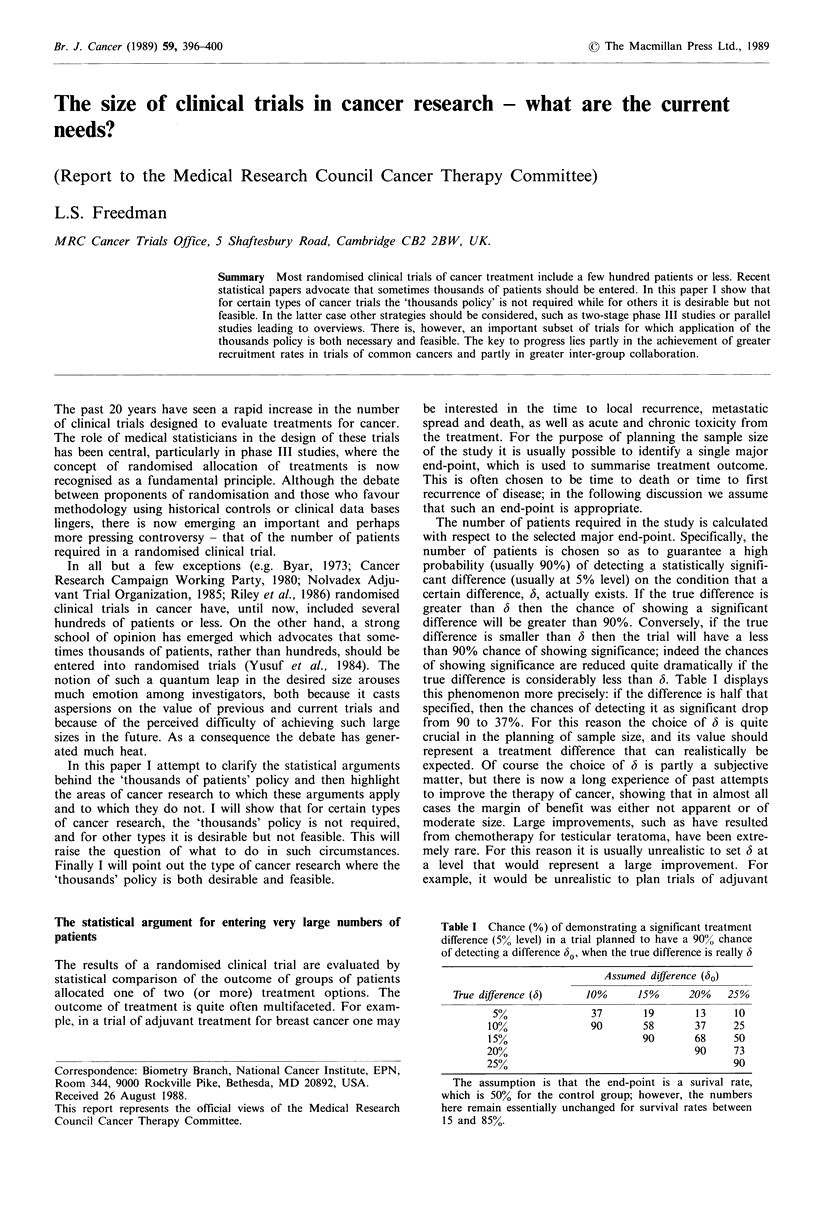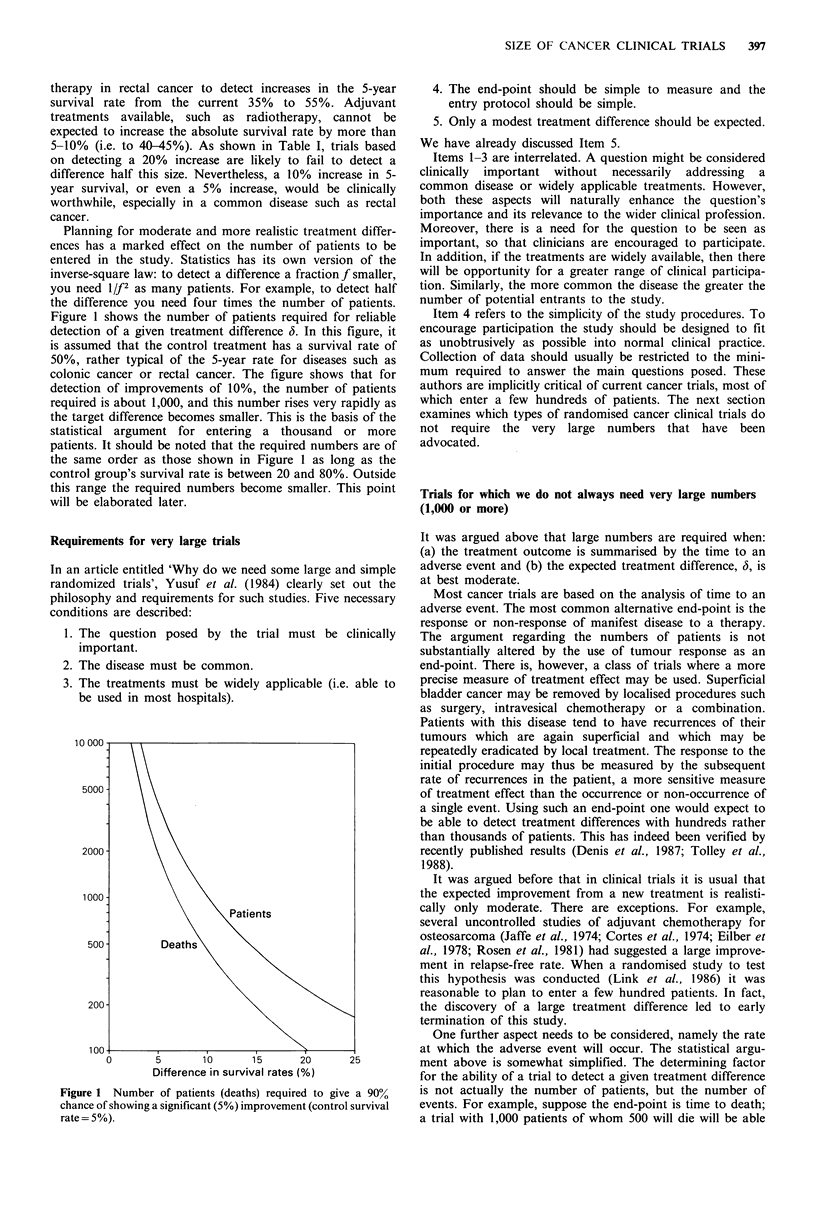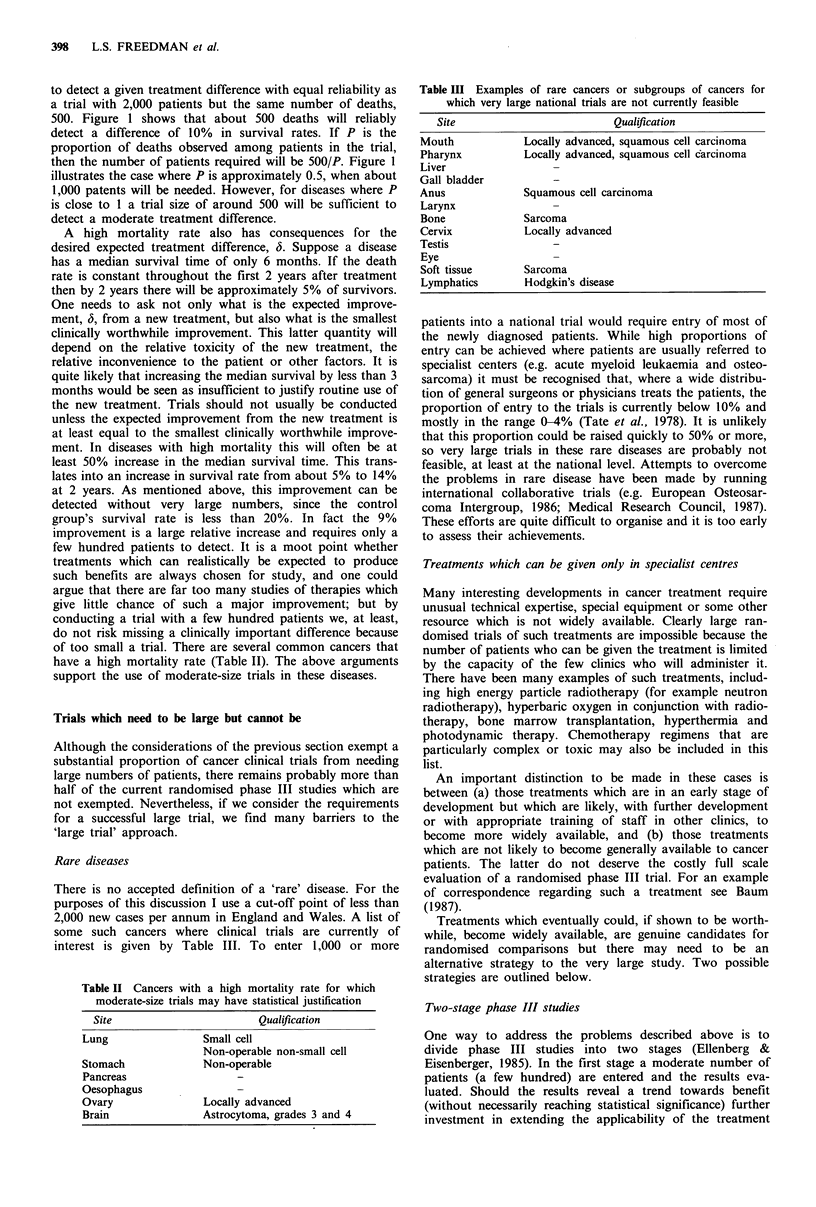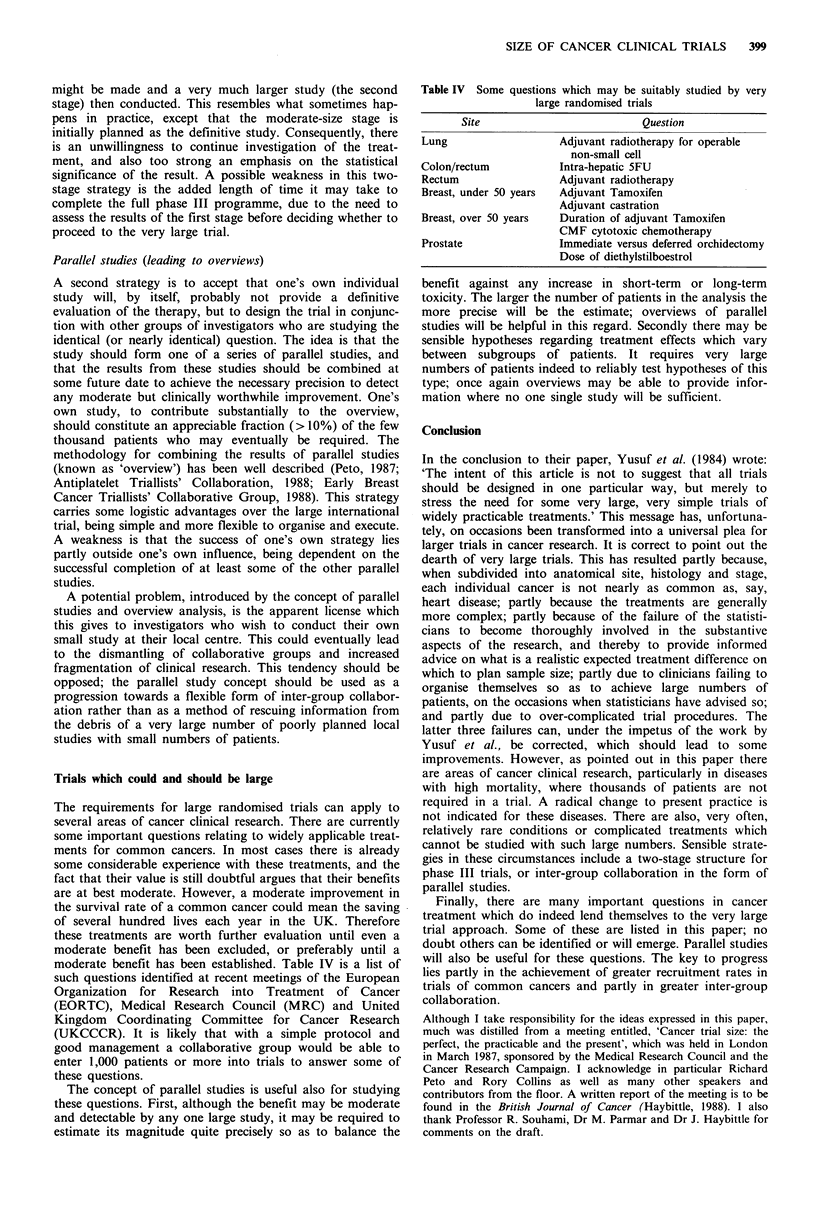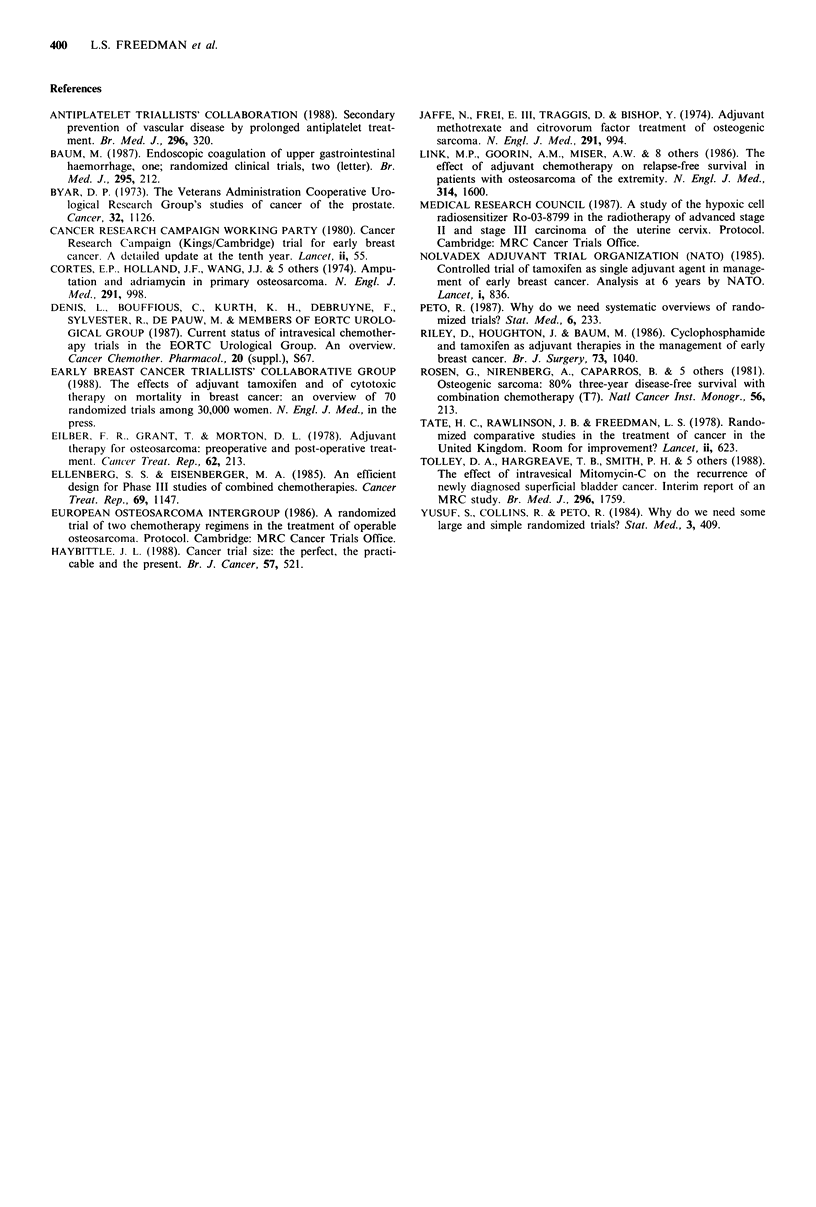# The size of clinical trials in cancer research--what are the current needs? Medical Research Council Cancer Therapy Committee.

**DOI:** 10.1038/bjc.1989.79

**Published:** 1989-03

**Authors:** L. S. Freedman

**Affiliations:** MRC Cancer Trials Office, Cambridge, UK.

## Abstract

Most randomised clinical trials of cancer treatment include a few hundred patients or less. Recent statistical papers advocate that sometimes thousands of patients should be entered. In this paper I show that for certain types of cancer trials the 'thousands policy' is not required while for others it is desirable but not feasible. In the latter case other strategies should be considered, such as two-stage phase III studies or parallel studies leading to overviews. There is, however, an important subset of trials for which application of the thousands policy is both necessary and feasible. The key to progress lies partly in the achievement of greater recruitment rates in trails of common cancers and partly in greater inter-group collaboration.


*Br. J. Cancer* (1989) **59**, 396–400 © The Macmillan Press Ltd., 1989

### The size of clinical trials in cancer research – what are the current needs?

### (Report to the Medical Research Council Cancer Therapy Committee)

L.S. Freedman

*MRC Cancer Trials Office, 5 Shaftesbury Road, Cambridge CB2 2BW, UK.*

**Summary** Most randomised clinical trials of cancer treatment include a few hundred patients or less. Recent statistical papers advocate that sometimes thousands of patients should be entered. In this paper I show that for certain types of cancer trials the 'thousands policy' is not required while for others it is desirable but not feasible. In the latter case other strategies should be considered, such as two-stage phase III studies or parallel studies leading to overviews. There is, however, an important subset of trials for which application of the thousands policy is both necessary and feasible. The key to progress lies partly in the achievement of greater recruitment rates in trials of common cancers and partly in greater inter-group collaboration.

The past 20 years have seen a rapid increase in the number of clinical trials designed to evaluate treatments for cancer. The role of medical statisticians in the design of these trials has been central, particularly in phase III studies, where the concept of randomised allocation of treatments is now recognised as a fundamental principle. Although the debate between proponents of randomisation and those who favour methodology using historical controls or clinical data bases lingers, there is now emerging an important and perhaps more pressing controversy – that of the number of patients required in a randomised clinical trial.

In all but a few exceptions (e.g. Byar, 1973; Cancer Research Campaign Working Party, 1980; Nolvadex Adjuvant Trial Organization, 1985; Riley *et al.*, 1986) randomised clinical trials in cancer have, until now, included several hundreds of patients or less. On the other hand, a strong school of opinion has emerged which advocates that sometimes thousands of patients, rather than hundreds, should be entered into randomised trials (Yusuf *et al.*, 1984). The notion of such a quantum leap in the desired size arouses much emotion among investigators, both because it casts aspersions on the value of previous and current trials and because of the perceived difficulty of achieving such large sizes in the future. As a consequence the debate has generated much heat.

In this paper I attempt to clarify the statistical arguments behind the 'thousands of patients' policy and then highlight the areas of cancer research to which these arguments apply and to which they do not. I will show that for certain types of cancer research, the 'thousands' policy is not required, and for other types it is desirable but not feasible. This will raise the question of what to do in such circumstances. Finally I will point out the type of cancer research where the 'thousands' policy is both desirable and feasible.

### The statistical argument for entering very large numbers of patients

The results of a randomised clinical trial are evaluated by statistical comparison of the outcome of groups of patients allocated one of two (or more) treatment options. The outcome of treatment is quite often multifaceted. For example, in a trial of adjuvant treatment for breast cancer one may be interested in the time to local recurrence, metastatic spread and death, as well as acute and chronic toxicity from the treatment. For the purpose of planning the sample size of the study it is usually possible to identify a single major end-point, which is used to summarise treatment outcome. This is often chosen to be time to death or time to first recurrence of disease; in the following discussion we assume that such an end-point is appropriate.

The number of patients required in the study is calculated with respect to the selected major end-point. Specifically, the number of patients is chosen so as to guarantee a high probability (usually 90%) of detecting a statistically significant difference (usually at 5% level) on the condition that a certain difference, $\delta$, actually exists. If the true difference is greater than $\delta$ then the chance of showing a significant difference will be greater than 90%. Conversely, if the true difference is smaller than $\delta$ then the trial will have a less than 90% chance of showing significance; indeed the chances of showing significance are reduced quite dramatically if the true difference is considerably less than $\delta$. Table I displays this phenomenon more precisely: if the difference is half that specified, then the chances of detecting it as significant drop from 90 to 37%. For this reason the choice of $\delta$ is quite crucial in the planning of sample size, and its value should represent a treatment difference that can realistically be expected. Of course the choice of $\delta$ is partly a subjective matter, but there is now a long experience of past attempts to improve the therapy of cancer, showing that in almost all cases the margin of benefit was either not apparent or of moderate size. Large improvements, such as have resulted from chemotherapy for testicular teratoma, have been extremely rare. For this reason it is usually unrealistic to set $\delta$ at a level that would represent a large improvement. For example, it would be unrealistic to plan trials of adjuvant

**Table I** Chance (%) of demonstrating a significant treatment difference (5% level) in a trial planned to have a 90% chance of detecting a difference $\delta_0$, when the true difference is really $\delta$

| | *Assumed difference* ($\delta_0$) | | | |
|---|---|---|---|---|
| *True difference* ($\delta$) | *10%* | *15%* | *20%* | *25%* |
| 5% | 37 | 19 | 13 | 10 |
| 10% | 90 | 58 | 37 | 25 |
| 15% | | 90 | 68 | 50 |
| 20% | | | 90 | 73 |
| 25% | | | | 90 |

The assumption is that the end-point is a surival rate, which is 50% for the control group; however, the numbers here remain essentially unchanged for survival rates between 15 and 85%.

Correspondence: Biometry Branch, National Cancer Institute, EPN, Room 344, 9000 Rockville Pike, Bethesda, MD 20892, USA.
Received 26 August 1988.
This report represents the official views of the Medical Research Council Cancer Therapy Committee.

therapy in rectal cancer to detect increases in the 5-year survival rate from the current 35% to 55%. Adjuvant treatments available, such as radiotherapy, cannot be expected to increase the absolute survival rate by more than 5–10% (i.e. to 40–45%). As shown in Table I, trials based on detecting a 20% increase are likely to fail to detect a difference half this size. Nevertheless, a 10% increase in 5-year survival, or even a 5% increase, would be clinically worthwhile, especially in a common disease such as rectal cancer.

Planning for moderate and more realistic treatment differences has a marked effect on the number of patients to be entered in the study. Statistics has its own version of the inverse-square law: to detect a difference a fraction $f$ smaller, you need $1/f^2$ as many patients. For example, to detect half the difference you need four times the number of patients. Figure 1 shows the number of patients required for reliable detection of a given treatment difference $\delta$. In this figure, it is assumed that the control treatment has a survival rate of 50%, rather typical of the 5-year rate for diseases such as colonic cancer or rectal cancer. The figure shows that for detection of improvements of 10%, the number of patients required is about 1,000, and this number rises very rapidly as the target difference becomes smaller. This is the basis of the statistical argument for entering a thousand or more patients. It should be noted that the required numbers are of the same order as those shown in Figure 1 as long as the control group's survival rate is between 20 and 80%. Outside this range the required numbers become smaller. This point will be elaborated later.

### Requirements for very large trials

In an article entitled 'Why do we need some large and simple randomized trials', Yusuf *et al.* (1984) clearly set out the philosophy and requirements for such studies. Five necessary conditions are described:

1. The question posed by the trial must be clinically important.
2. The disease must be common.
3. The treatments must be widely applicable (i.e. able to be used in most hospitals).

**Figure 1** Number of patients (deaths) required to give a 90% chance of showing a significant (5%) improvement (control survival rate = 5%).

4. The end-point should be simple to measure and the entry protocol should be simple.
5. Only a modest treatment difference should be expected.

We have already discussed Item 5.

Items 1–3 are interrelated. A question might be considered clinically important without necessarily addressing a common disease or widely applicable treatments. However, both these aspects will naturally enhance the question's importance and its relevance to the wider clinical profession. Moreover, there is a need for the question to be seen as important, so that clinicians are encouraged to participate. In addition, if the treatments are widely available, then there will be opportunity for a greater range of clinical participation. Similarly, the more common the disease the greater the number of potential entrants to the study.

Item 4 refers to the simplicity of the study procedures. To encourage participation the study should be designed to fit as unobtrusively as possible into normal clinical practice. Collection of data should usually be restricted to the minimum required to answer the main questions posed. These authors are implicitly critical of current cancer trials, most of which enter a few hundreds of patients. The next section examines which types of randomised cancer clinical trials do not require the very large numbers that have been advocated.

### Trials for which we do not always need very large numbers (1,000 or more)

It was argued above that large numbers are required when: (a) the treatment outcome is summarised by the time to an adverse event and (b) the expected treatment difference, $\delta$, is at best moderate.

Most cancer trials are based on the analysis of time to an adverse event. The most common alternative end-point is the response or non-response of manifest disease to a therapy. The argument regarding the numbers of patients is not substantially altered by the use of tumour response as an end-point. There is, however, a class of trials where a more precise measure of treatment effect may be used. Superficial bladder cancer may be removed by localised procedures such as surgery, intravesical chemotherapy or a combination. Patients with this disease tend to have recurrences of their tumours which are again superficial and which may be repeatedly eradicated by local treatment. The response to the initial procedure may thus be measured by the subsequent rate of recurrences in the patient, a more sensitive measure of treatment effect than the occurrence or non-occurrence of a single event. Using such an end-point one would expect to be able to detect treatment differences with hundreds rather than thousands of patients. This has indeed been verified by recently published results (Denis *et al.*, 1987; Tolley *et al.*, 1988).

It was argued before that in clinical trials it is usual that the expected improvement from a new treatment is realistically only moderate. There are exceptions. For example, several uncontrolled studies of adjuvant chemotherapy for osteosarcoma (Jaffe *et al.*, 1974; Cortes *et al.*, 1974; Eilber *et al.*, 1978; Rosen *et al.*, 1981) had suggested a large improvement in relapse-free rate. When a randomised study to test this hypothesis was conducted (Link *et al.*, 1986) it was reasonable to plan to enter a few hundred patients. In fact, the discovery of a large treatment difference led to early termination of this study.

One further aspect needs to be considered, namely the rate at which the adverse event will occur. The statistical argument above is somewhat simplified. The determining factor for the ability of a trial to detect a given treatment difference is not actually the number of patients, but the number of events. For example, suppose the end-point is time to death; a trial with 1,000 patients of whom 500 will die will be able to detect a given treatment difference with equal reliability as a trial with 2,000 patients but the same number of deaths, 500. Figure 1 shows that about 500 deaths will reliably detect a difference of 10% in survival rates. If $P$ is the proportion of deaths observed among patients in the trial, then the number of patients required will be $500/P$. Figure 1 illustrates the case where $P$ is approximately 0.5, when about 1,000 patents will be needed. However, for diseases where $P$ is close to 1 a trial size of around 500 will be sufficient to detect a moderate treatment difference.

A high mortality rate also has consequences for the desired expected treatment difference, $\delta$. Suppose a disease has a median survival time of only 6 months. If the death rate is constant throughout the first 2 years after treatment then by 2 years there will be approximately 5% of survivors. One needs to ask not only what is the expected improvement, $\delta$, from a new treatment, but also what is the smallest clinically worthwhile improvement. This latter quantity will depend on the relative toxicity of the new treatment, the relative inconvenience to the patient or other factors. It is quite likely that increasing the median survival by less than 3 months would be seen as insufficient to justify routine use of the new treatment. Trials should not usually be conducted unless the expected improvement from the new treatment is at least equal to the smallest clinically worthwhile improvement. In diseases with high mortality this will often be at least 50% increase in the median survival time. This translates into an increase in survival rate from about 5% to 14% at 2 years. As mentioned above, this improvement can be detected without very large numbers, since the control group's survival rate is less than 20%. In fact the 9% improvement is a large relative increase and requires only a few hundred patients to detect. It is a moot point whether treatments which can realistically be expected to produce such benefits are always chosen for study, and one could argue that there are far too many studies of therapies which give little chance of such a major improvement; but by conducting a trial with a few hundred patients we, at least, do not risk missing a clinically important difference because of too small a trial. There are several common cancers that have a high mortality rate (Table II). The above arguments support the use of moderate-size trials in these diseases.

### Trials which need to be large but cannot be

Although the considerations of the previous section exempt a substantial proportion of cancer clinical trials from needing large numbers of patients, there remains probably more than half of the current randomised phase III studies which are not exempted. Nevertheless, if we consider the requirements for a successful large trial, we find many barriers to the 'large trial' approach.

### *Rare diseases*

There is no accepted definition of a 'rare' disease. For the purposes of this discussion I use a cut-off point of less than 2,000 new cases per annum in England and Wales. A list of some such cancers where clinical trials are currently of interest is given by Table III. To enter 1,000 or more

**Table II** Cancers with a high mortality rate for which moderate-size trials may have statistical justification

| *Site* | *Qualification* |
|---|---|
| Lung | Small cell |
| | Non-operable non-small cell |
| Stomach | Non-operable |
| Pancreas | – |
| Oesophagus | – |
| Ovary | Locally advanced |
| Brain | Astrocytoma, grades 3 and 4 |

**Table III** Examples of rare cancers or subgroups of cancers for which very large national trials are not currently feasible

| *Site* | *Qualification* |
|---|---|
| Mouth | Locally advanced, squamous cell carcinoma |
| Pharynx | Locally advanced, squamous cell carcinoma |
| Liver | – |
| Gall bladder | – |
| Anus | Squamous cell carcinoma |
| Larynx | – |
| Bone | Sarcoma |
| Cervix | Locally advanced |
| Testis | – |
| Eye | – |
| Soft tissue | Sarcoma |
| Lymphatics | Hodgkin's disease |

patients into a national trial would require entry of most of the newly diagnosed patients. While high proportions of entry can be achieved where patients are usually referred to specialist centers (e.g. acute myeloid leukaemia and osteosarcoma) it must be recognised that, where a wide distribution of general surgeons or physicians treats the patients, the proportion of entry to the trials is currently below 10% and mostly in the range 0–4% (Tate *et al.*, 1978). It is unlikely that this proportion could be raised quickly to 50% or more, so very large trials in these rare diseases are probably not feasible, at least at the national level. Attempts to overcome the problems in rare disease have been made by running international collaborative trials (e.g. European Osteosarcoma Intergroup, 1986; Medical Research Council, 1987). These efforts are quite difficult to organise and it is too early to assess their achievements.

### *Treatments which can be given only in specialist centres*

Many interesting developments in cancer treatment require unusual technical expertise, special equipment or some other resource which is not widely available. Clearly large randomised trials of such treatments are impossible because the number of patients who can be given the treatment is limited by the capacity of the few clinics who will administer it. There have been many examples of such treatments, including high energy particle radiotherapy (for example neutron radiotherapy), hyperbaric oxygen in conjunction with radiotherapy, bone marrow transplantation, hyperthermia and photodynamic therapy. Chemotherapy regimens that are particularly complex or toxic may also be included in this list.

An important distinction to be made in these cases is between (a) those treatments which are in an early stage of development but which are likely, with further development or with appropriate training of staff in other clinics, to become more widely available, and (b) those treatments which are not likely to become generally available to cancer patients. The latter do not deserve the costly full scale evaluation of a randomised phase III trial. For an example of correspondence regarding such a treatment see Baum (1987).

Treatments which eventually could, if shown to be worthwhile, become widely available, are genuine candidates for randomised comparisons but there may need to be an alternative strategy to the very large study. Two possible strategies are outlined below.

### *Two-stage phase III studies*

One way to address the problems described above is to divide phase III studies into two stages (Ellenberg & Eisenberger, 1985). In the first stage a moderate number of patients (a few hundred) are entered and the results evaluated. Should the results reveal a trend towards benefit (without necessarily reaching statistical significance) further investment in extending the applicability of the treatment might be made and a very much larger study (the second stage) then conducted. This resembles what sometimes happens in practice, except that the moderate-size stage is initially planned as the definitive study. Consequently, there is an unwillingness to continue investigation of the treatment, and also too strong an emphasis on the statistical significance of the result. A possible weakness in this two-stage strategy is the added length of time it may take to complete the full phase III programme, due to the need to assess the results of the first stage before deciding whether to proceed to the very large trial.

*Parallel studies (leading to overviews)*

A second strategy is to accept that one's own individual study will, by itself, probably not provide a definitive evaluation of the therapy, but to design the trial in conjunction with other groups of investigators who are studying the identical (or nearly identical) question. The idea is that the study should form one of a series of parallel studies, and that the results from these studies should be combined at some future date to achieve the necessary precision to detect any moderate but clinically worthwhile improvement. One's own study, to contribute substantially to the overview, should constitute an appreciable fraction (>10%) of the few thousand patients who may eventually be required. The methodology for combining the results of parallel studies (known as 'overview') has been well described (Peto, 1987; Antiplatelet Triallists' Collaboration, 1988; Early Breast Cancer Triallists' Collaborative Group, 1988). This strategy carries some logistic advantages over the large international trial, being simple and more flexible to organise and execute. A weakness is that the success of one's own strategy lies partly outside one's own influence, being dependent on the successful completion of at least some of the other parallel studies.

A potential problem, introduced by the concept of parallel studies and overview analysis, is the apparent license which this gives to investigators who wish to conduct their own small study at their local centre. This could eventually lead to the dismantling of collaborative groups and increased fragmentation of clinical research. This tendency should be opposed; the parallel study concept should be used as a progression towards a flexible form of inter-group collaboration rather than as a method of rescuing information from the debris of a very large number of poorly planned local studies with small numbers of patients.

### Trials which could and should be large

The requirements for large randomised trials can apply to several areas of cancer clinical research. There are currently some important questions relating to widely applicable treatments for common cancers. In most cases there is already some considerable experience with these treatments, and the fact that their value is still doubtful argues that their benefits are at best moderate. However, a moderate improvement in the survival rate of a common cancer could mean the saving of several hundred lives each year in the UK. Therefore these treatments are worth further evaluation until even a moderate benefit has been excluded, or preferably until a moderate benefit has been established. Table IV is a list of such questions identified at recent meetings of the European Organization for Research into Treatment of Cancer (EORTC), Medical Research Council (MRC) and United Kingdom Coordinating Committee for Cancer Research (UKCCCR). It is likely that with a simple protocol and good management a collaborative group would be able to enter 1,000 patients or more into trials to answer some of these questions.

The concept of parallel studies is useful also for studying these questions. First, although the benefit may be moderate and detectable by any one large study, it may be required to estimate its magnitude quite precisely so as to balance the

**Table IV** Some questions which may be suitably studied by very large randomised trials

| *Site* | *Question* |
|---|---|
| Lung | Adjuvant radiotherapy for operable non-small cell |
| Colon/rectum | Intra-hepatic 5FU |
| Rectum | Adjuvant radiotherapy |
| Breast, under 50 years | Adjuvant Tamoxifen |
| | Adjuvant castration |
| Breast, over 50 years | Duration of adjuvant Tamoxifen |
| | CMF cytotoxic chemotherapy |
| Prostate | Immediate versus deferred orchidectomy |
| | Dose of diethylstilboestrol |

benefit against any increase in short-term or long-term toxicity. The larger the number of patients in the analysis the more precise will be the estimate; overviews of parallel studies will be helpful in this regard. Secondly there may be sensible hypotheses regarding treatment effects which vary between subgroups of patients. It requires very large numbers of patients indeed to reliably test hypotheses of this type; once again overviews may be able to provide information where no one single study will be sufficient.

### Conclusion

In the conclusion to their paper, Yusuf *et al.* (1984) wrote: 'The intent of this article is not to suggest that all trials should be designed in one particular way, but merely to stress the need for some very large, very simple trials of widely practicable treatments.' This message has, unfortunately, on occasions been transformed into a universal plea for larger trials in cancer research. It is correct to point out the dearth of very large trials. This has resulted partly because, when subdivided into anatomical site, histology and stage, each individual cancer is not nearly as common as, say, heart disease; partly because the treatments are generally more complex; partly because of the failure of the statisticians to become thoroughly involved in the substantive aspects of the research, and thereby to provide informed advice on what is a realistic expected treatment difference on which to plan sample size; partly due to clinicians failing to organise themselves so as to achieve large numbers of patients, on the occasions when statisticians have advised so; and partly due to over-complicated trial procedures. The latter three failures can, under the impetus of the work by Yusuf *et al.*, be corrected, which should lead to some improvements. However, as pointed out in this paper there are areas of cancer clinical research, particularly in diseases with high mortality, where thousands of patients are not required in a trial. A radical change to present practice is not indicated for these diseases. There are also, very often, relatively rare conditions or complicated treatments which cannot be studied with such large numbers. Sensible strategies in these circumstances include a two-stage structure for phase III trials, or inter-group collaboration in the form of parallel studies.

Finally, there are many important questions in cancer treatment which do indeed lend themselves to the very large trial approach. Some of these are listed in this paper; no doubt others can be identified or will emerge. Parallel studies will also be useful for these questions. The key to progress lies partly in the achievement of greater recruitment rates in trials of common cancers and partly in greater inter-group collaboration.

Although I take responsibility for the ideas expressed in this paper, much was distilled from a meeting entitled, 'Cancer trial size: the perfect, the practicable and the present', which was held in London in March 1987, sponsored by the Medical Research Council and the Cancer Research Campaign. I acknowledge in particular Richard Peto and Rory Collins as well as many other speakers and contributors from the floor. A written report of the meeting is to be found in the *British Journal of Cancer* (Haybittle, 1988). I also thank Professor R. Souhami, Dr M. Parmar and Dr J. Haybittle for comments on the draft.

### References

ANTIPLATELET TRIALLISTS' COLLABORATION (1988). Secondary prevention of vascular disease by prolonged antiplatelet treatment. *Br. Med. J.*, **296**, 320.

BAUM, M. (1987). Endoscopic coagulation of upper gastrointestinal haemorrhage, one; randomized clinical trials, two (letter). *Br. Med. J.*, **295**, 212.

BYAR, D. P. (1973). The Veterans Administration Cooperative Urological Research Group's studies of cancer of the prostate. *Cancer*, **32**, 1126.

CANCER RESEARCH CAMPAIGN WORKING PARTY (1980). Cancer Research Campaign (Kings/Cambridge) trial for early breast cancer. A detailed update at the tenth year. *Lancet*, **ii**, 55.

CORTES, E.P., HOLLAND, J.F., WANG, J.J. & 5 others (1974). Amputation and adriamycin in primary osteosarcoma. *N. Engl. J. Med.*, **291**, 998.

DENIS, L., BOUFFIOUS, C., KURTH, K. H., DEBRUYNE, F., SYLVESTER, R., DE PAUW, M. & MEMBERS OF EORTC UROLOGICAL GROUP (1987). Current status of intravesical chemotherapy trials in the EORTC Urological Group. An overview. *Cancer Chemother. Pharmacol.*, **20** (suppl.), S67.

EARLY BREAST CANCER TRIALLISTS' COLLABORATIVE GROUP (1988). The effects of adjuvant tamoxifen and of cytotoxic therapy on mortality in breast cancer: an overview of 70 randomized trials among 30,000 women. *N. Engl. J. Med.*, in the press.

EILBER, F. R., GRANT, T. & MORTON, D. L. (1978). Adjuvant therapy for osteosarcoma: preoperative and post-operative treatment. *Cancer Treat. Rep.*, **62**, 213.

ELLENBERG, S. S. & EISENBERGER, M. A. (1985). An efficient design for Phase III studies of combined chemotherapies. *Cancer Treat. Rep.*, **69**, 1147.

EUROPEAN OSTEOSARCOMA INTERGROUP (1986). A randomized trial of two chemotherapy regimens in the treatment of operable osteosarcoma. Protocol. Cambridge: MRC Cancer Trials Office.

HAYBITTLE, J. L. (1988). Cancer trial size: the perfect, the practicable and the present. *Br. J. Cancer*, **57**, 521.

JAFFE, N., FREI, E. III, TRAGGIS, D. & BISHOP, Y. (1974). Adjuvant methotrexate and citrovorum factor treatment of osteogenic sarcoma. *N. Engl. J. Med.*, **291**, 994.

LINK, M.P., GOORIN, A.M., MISER, A.W. & 8 others (1986). The effect of adjuvant chemotherapy on relapse-free survival in patients with osteosarcoma of the extremity. *N. Engl. J. Med.*, **314**, 1600.

MEDICAL RESEARCH COUNCIL (1987). A study of the hypoxic cell radiosensitizer Ro-03-8799 in the radiotherapy of advanced stage II and stage III carcinoma of the uterine cervix. Protocol. Cambridge: MRC Cancer Trials Office.

NOLVADEX ADJUVANT TRIAL ORGANIZATION (NATO) (1985). Controlled trial of tamoxifen as single adjuvant agent in management of early breast cancer. Analysis at 6 years by NATO. *Lancet*, **i**, 836.

PETO, R. (1987). Why do we need systematic overviews of randomized trials? *Stat. Med.*, **6**, 233.

RILEY, D., HOUGHTON, J. & BAUM, M. (1986). Cyclophosphamide and tamoxifen as adjuvant therapies in the management of early breast cancer. *Br. J. Surgery*, **73**, 1040.

ROSEN, G., NIRENBERG, A., CAPARROS, B. & 5 others (1981). Osteogenic sarcoma: 80% three-year disease-free survival with combination chemotherapy (T7). *Natl Cancer Inst. Monogr.*, **56**, 213.

TATE, H. C., RAWLINSON, J. B. & FREEDMAN, L. S. (1978). Randomized comparative studies in the treatment of cancer in the United Kingdom. Room for improvement? *Lancet*, **ii**, 623.

TOLLEY, D. A., HARGREAVE, T. B., SMITH, P. H. & 5 others (1988). The effect of intravesical Mitomycin-C on the recurrence of newly diagnosed superficial bladder cancer. Interim report of an MRC study. *Br. Med. J.*, **296**, 1759.

YUSUF, S., COLLINS, R. & PETO, R. (1984). Why do we need some large and simple randomized trials? *Stat. Med.*, **3**, 409.